# SOX2 and SOX2-MYC Reprogramming Process of Fibroblasts to the Neural Stem Cells Compromised by Senescence

**DOI:** 10.1371/journal.pone.0141688

**Published:** 2015-11-04

**Authors:** Marta Winiecka-Klimek, Maciej Smolarz, Maciej P. Walczak, Jolanta Zieba, Krystyna Hulas-Bigoszewska, Blazej Kmieciak, Sylwester Piaskowski, Piotr Rieske, Dawid P. Grzela, Ewelina Stoczynska-Fidelus

**Affiliations:** 1 Department of Research and Development, Celther Polska, Lodz, Poland; 2 Department of Tumor Biology, Medical University of Lodz, Lodz, Poland; 3 Department of Medical Law, Chair of Human Sciences, Medical University of Lodz, Lodz, Poland; University of Kansas Medical Center, UNITED STATES

## Abstract

Tumorigenic potential of induced pluripotent stem cells (iPSCs) infiltrating population of induced neural stem cells (iNSCs) generated from iPSCs may limit their medical applications. To overcome such a difficulty, direct reprogramming of adult somatic cells into iNSCs was proposed. The aim of this study was the systematic comparison of induced neural cells (iNc) obtained with different methods—direct reprogramming of human adult fibroblasts with either SOX2 (SiNSc-like) or SOX2 and c-MYC (SMiNSc-like) and induced pluripotent stem cells differentiation to ebiNSc—in terms of gene expression profile, differentiation potential as well as proliferation properties. Immunocytochemistry and real-time PCR analyses were used to evaluate gene expression profile and differentiation potential of various iNc types. Bromodeoxyuridine (BrdU) incorporation and senescence-associated beta-galactosidase (SA-β-gal) assays were used to estimate proliferation potential. All three types of iNc were capable of neuronal differentiation; however, astrocytic differentiation was possible only in case of ebiNSc. Contrary to ebiNSc generation, the direct reprogramming was rarely a propitious process, despite 100% transduction efficiency. The potency of direct iNSCs-like cells generation was lower as compared to iNSCs obtained by iPSCs differentiation, and only slightly improved when c-MYC was added. Directly reprogrammed iNSCs-like cells were lacking the ability to differentiate into astrocytic cells and characterized by poor efficiency of neuronal cells formation. Such features indicated that these cells could not be fully reprogrammed, as confirmed mainly with senescence detection. Importantly, SiNSc-like and SMiNSc-like cells were unable to achieve the long-term survival and became senescent, which limits their possible therapeutic applicability. Our results suggest that iNSCs-like cells, generated in the direct reprogramming attempts, were either not fully reprogrammed or reprogrammed only into neuronal progenitors, mainly because of the inaccuracies of currently available protocols.

## Introduction

Recent progress made in the field of nuclear reprogramming and differentiation of stem cells provides a great tool that can be applied to modeling of various human diseases as well as to regenerative medicine. Nonetheless, current treatment of various neurological disorders including neurodegenerative diseases such as Alzheimer’s disease (AD) or Parkinson’s disease (PD) is not efficient enough. So far, several methods for generation of specialized neurons, i.a. dopaminergic [1,2] or functional spinal motor neurons [3], from mouse and human fibroblast have been developed. The aforementioned issues are hoped to be solved thanks to application of stem cells technology that involves generation of induced neural stem cells (iNSCs) from differentiated somatic cells. Nowadays, two methods of iNSCs generation are available: direct reprogramming of somatic cells without going through the pluripotent state or with the generation of iPSCs as an intermediate step [4–8]. Although the tumorigenic potential of iPSCs may be debatable, all *in vivo* studies, in which iNSCs were obtained with the intermediate iPSCs step, report the lack of tumorigenic outgrowth [9]. Even though the second approach seems to be very promising in regard to regenerative medicine, there are still many obstacles, which need to be overcome in order to enable generation of mature and fully functional neurons. Very low efficiency of direct reprogramming, ranging from 0.009–0.96% [7,10–12], constitutes one of the major problems. Moreover, although it was shown that iNSCs are capable of differentiating into functionally mature neurons and GFAP-expressing cells, their oligodendrocytic differentiation still remains challenging [6,13].

For many years reprogramming process with only one transcription factor has been considered insufficient for neural stem cells formation [6]. However, in 2012 Ring *et al*. demonstrated that under conditions conducive to neural stem cell expansion, including the presence of growth factors and proper surface substrates, overexpression of SOX2 is enough to reprogram fibroblasts into multipotent neural stem cells [7]. Furthermore, it has been shown that endogenous expression of neural precursor genes such as *SOX1*, *SOX3*, *OLIG2*, *NCAM* and *PAX6* can be detected only in the presence of SOX2 [13]. Hence, this transcription factor is thought to play a critical role in direct reprogramming, which probably may be explained by the fact that SOX2 is known to activate many genes that overlap the targeted genes for OCT4 or NANOG [14].

The reprogramming efficiency, defined as the colony formation in relation to the number of fibroblasts initially transfected/transduced, is widely analyzed. However, the proliferation potential is omitted in the majority of studies. It needs to be emphasized that if human neural stem cells proliferate infinitely, the efficiency of reprogramming process will be less important. To increase the proliferation potential and to enable extensive passaging of non-viral generated iNSCs Maucksch *et al*. transfected fibroblasts with SOX2 and PAX6 [11]. The latter factor is essential for neural stem cells proliferation, multipotency and neurogenesis [15]. Nonetheless, proliferation potential, in contrast to multipotency and electrophysiology, is still poorly studied. Unlike human iNSCs, mouse iNSCs are widely described as easily propagated, even up to 30 passages [7].

In this study, we provide the first description of direct reprogramming of human adult fibroblasts using SOX2 and c-MYC, performed to optimize proliferation capabilities of induced neural stem cells-like cells (iNSCs-like cells). Quality and efficiency of iNSCs-like cells generation by means of different methods were compared. In our work, iNc were obtained either by differentiation of induced pluripotent stem cells or by direct reprograming with one (SOX2) or two transcription factors (SOX2 and c-MYC). SiNSc-like and SMiNSc-like cells were compared to ebiNSc and neural stem cells (NSC) derived from human embryonic stem cells (hESCs) in terms of gene expression profile, differentiation potential and proliferation properties. Finally, proliferation analyses were conducted to verify whether direct reprogramming of adult fibroblasts into iNSCs-like cells is able to provide a virtually limitless source of specific neural cells applicable in many medical areas, such as regenerative therapy or modeling of various neurological disorders (e.g. Alzheimer’s or Parkinson’s diseases).

## Materials and Methods

### Reprogramming of the BJ fibroblasts into iPSCs

In Celther Polska laboratories induced pluripotent stem cells are generated either by lentiviral transduction or by episomal transfection [16]. For this study, human iPSCs were generated from BJ human neonatal foreskin fibroblasts (ATCC, USA) (at passages 1–3) by lentiviral transduction using polycistronic vector pLV-OSKM containing four transcription factors—OCT4, SOX2, KLF4 and c-MYC (LENTI-Smart OSKM kit, InvivoGen, France). One day prior to transduction BJ cells were plated at 5 × 10^5^ cells/well density on a 6-well plate in DMEM supplemented with 10% FBS (Life Technologies, Thermo Fisher Scientific, USA) and antibiotics (penicillin/streptomycin/gentamicin; GIBCO, Thermo Fisher Scientific). Next day lentiviral medium, which was collected from transfected HEK293T cells (ATCC), filtered through a 0.45 μm filter (Merck Millipore, Germany) and supplemented with 8 μg/ml of Polybrene (Merck Millipore), was added to BJ culture. The following day second lentiviral transduction was performed. After 24 hours medium was changed to fibroblast culture medium. Seven days after transduction, reprogrammed cells were seeded in ESC medium (DMEM/F-12 with GlutaMAX-I (1X), KnockOut Serum Replacement 20%, 2-Mercaptoethanol 100 μM, all from Life Technologies, Non Essential Amino Acids 1% from Gibco and bFGF 10 ng/ml from Sigma Aldrich, USA) on 10 cm culture dishes coated with feeder layer– 1.5 × 10^6^ Mouse Embryonic Fibroblasts inactivated with Mitomycin C (Merck Millipore). After another 7–14 days formed ES-like colonies were collected and subsequently cultured on Geltrex-coated dishes in the Essential 8 medium (both from Life Technologies).

### Differentiation of iPSCs into iNSCs

The protocol of iPSCs differentiation into iNSCs involving EB formation was based on the method presented by Yuan *et al*. [17]. The whole procedure can be divided into several distinct steps. At approximately 80% confluency iPSCs colonies were firstly transferred from 4 wells of 6-well cell culture plate (Nunc, Thermo Fisher Scientific) coated with Geltrex to a 6 cm non-adhesive culture dish/T25 non-adhesive culture bottle (Sarstedt, Germany) and grown in suspension culture in Embryoid Body Formation medium (DMEM/F-12 with GlutaMAX-I (1X), KnockOut Serum Replacement 20%, 2-Mercaptoethanol 100 μM, Non Essential Amino Acids 1%) for 4 days. During the next 4 days, 5 × 10^−7^ M retinoic acid (Sigma-Aldrich) was added daily to the suspension culture. Next, 5 to 10 EBs were plated on Poly-L-Ornithine (20 μg/ml)/Laminin (10 μg/ml) (both from Sigma Aldrich)–coated 6-well adherent culture dishes (Nunc) and grown in Neural Stem Cell Induction medium (DMEM/F12 1X, B-27 Supplement 2% both from Life Technologies), supplemented with 10 ng/ml hLIF (Merck Milipore), 2 μg/ml heparin sodium salt in PBS (STEMCELL Technologies, Canada), 20 ng/ml EGF (Sigma Aldrich), 10 ng/ml bFGF for the following 7 days. After reaching 85% confluency, 0.5–1 × 10^6^ cells were seeded on Geltrex-coated dishes with ReNcell medium (Merck Millipore) supplemented with 20 ng/ml EGF and 20 ng/ml bFGF.

### Reprogramming of BJ fibroblasts into SiNSc-like and SMiNSc-like cells

Human iNSCs-like cells were generated from BJ human neonatal foreskin fibroblasts (at passages 1–3) by lentiviral transduction using SOX2 alone or SOX2 and c-MYC. Reprogramming protocol, dedicated for iNSCs generation, was based on the method presented by Ring *et al*. [7] with some modifications. One day prior to transduction BJ human neonatal foreskin fibroblasts were seeded in 24-well plates (1 × 10^4^ cells/well) covered with feeder layer prepared the previous day– 1.25 × 10^5^ Mitomycin C-treated MEFs per well seeded on coverslips (Marienfeld Laboratory Glassware, Germany) coated with 0.1% gelatin. According to Ring *et al*., the presence of coverslips drastically alters transduced cells morphology and therefore increases the efficiency of iNSCs generation [7]. BJ cells were transduced with lentiviral medium, which was collected from transfected HEK293T cells, filtered through a 0.45 μm filter and supplemented with 8 μg/ml of Polybrene. Next day the procedure was repeated. Twenty-four hours post-transduction medium was changed to fibroblast culture medium and after following 5 days to Neural Stem Cell Culture medium (ReNcell or complete StemPro NSC Serum Free Medium, both supplemented with 20 ng/ml EGF and 20 ng/ml bFGF) and maintained in such conditions for another 7–10 days. iNSCs-like cells were subjected (mechanically or enzymatically—StemPro Accutase, Life Technologies) to propagation and rounds of neurosphere formation.

### Maintaining cell culture

The iPSCs were cultured on Geltrex-coated dishes in Essential 8 medium and passaged with 0.5 mM EDTA (Life Technologies) solution in PBS every 3–4 days.

BG01V, human embryonic stem cell line was purchased from AMS Biotechnology (Abingdon, UK). hESCs were cultured on Geltrex-coated dishes in Essential 8 medium and passaged with 0.5 mM EDTA solution in PBS every 3–4 days.

Human Neural Stem Cells (NSC; derived from the NIH approved H9 (WA09) human embryonic stem cells, hESCs) were purchased from Gibco. NSC cell line was propagated as an adherent culture on Geltrex-coated dishes in complete StemPro NSC SFM (KnockOut D-MEM/F-12, GlutaMAX-I Supplement 2 mM, StemPro Neural Supplement 2% from Life Technologies, bFGF 20 ng/ml, EGF 20 ng/ml). Culture medium was changed every two days and cells were passaged with StemPro Accutase when confluency reached 85%.

ebiNS cell line was propagated as an adherent culture on Geltrex-coated dishes in ReNcell medium supplemented with bFGF 20 ng/ml and EGF 20 ng/ml. Culture medium was changed every two days and cells were passaged with StemPro Accutase when confluency reached 85%.

SiNSc-like and SMiNSc-like cells were propagated as adherent cultures on Geltrex-coated dishes in ReNcell or complete StemPro NSC SFM medium supplemented with bFGF 20 ng/ml and EGF 20 ng/ml. Culture medium was changed every two days and cells were passaged with StemPro Accutase when confluency reached 85%.

### The efficiency of lentiviral transduction

The transduction efficiency was calculated as a ratio of SOX2-positive cells to the total number of BJ fibroblasts transduced with SOX2 or SOX2 and c-MYC. Immunocytochemical staining was performed with anti-SOX2 specific antibody four days after lentivirus delivery.

### The efficiency of obtaining induced Neural Cells

In order to compare the efficiency of obtaining induced neural cells either by direct reprogramming or iPSCs differentiation, the number of double SOX2- and nestin-positive cells (at the second passage of the culture) in at least ten randomly selected fields of view from three independent experiments was counted.

### Differentiation of Neural Cells (NSC, ebiNSc, SiNSc-like and SMiNSc-like cells) into neuronal and astrocytic cells

Cells were seeded at 2.5 × 10^4^ cells/well density onto 4-well plates (Nunc) with coverslips covered with Geltrex in medium for neural stem cell maintenance (ReNcell or StemPro NSC supplemented with bFGF and EGF). After 24 hours, medium was changed to neuronal differentiation medium (Neurobasal Medium 1X, B-27 Supplement 2%, GlutaMAX-I Supplement 2 mM all from Life Technologies). Half of medium was changed every 2–3 days. Cells were cultured in neural induction medium for 7 or 14 days, then subjected to immunocytochemical analysis.

### The potency of ebiNSc, SiNSc-like and SMiNSc-like cells differentiation

In order to compare the potency of ebiNSc, SiNSc-like and SMiNSc-like cells differentiation, the number of MAP2-positive cells after 7 days of differentiation, in at least ten randomly selected fields of view from three independent experiments was counted.

### RNA isolation

Total RNA was extracted using AllPrep DNA/RNA Mini Kit 50 (Qiagen, Germany) according to the manufacturer’s protocol. 250 ng of RNA was reverse transcribed to cDNA using QuantiTect Reverse Transcription Kit 50 (Qiagen) according to the manufacturer’s protocol. Samples for real-time PCR analysis were collected between 3^rd^-7^th^ week of direct reprogramming (iNSCs-like cells) and between 3^rd^-8^th^ passage (NSC, ebiNSc, hESCs).

### Real-Time PCR Analysis

Real-time PCR was performed using StepOnePlus Real-Time PCR System instrument (Applied Biosystems, Thermo Fisher Scientific). PCR products were synthesized from cDNA samples using SYBR^®^ Select Master Mix. Each sample was amplified in triplicate in total reaction volume of 12 μl containing SYBR^®^ Select Master Mix (2X) (Life Technologies), 200 nM of both forward and reverse primers and 250 ng of cDNA. *HPRT1* gene was used as a reference gene to normalize the expression levels of target gene, and tested genes were amplified using specific primers (S1 Table). Cycling conditions were as follows: 2 min at 50°C (UDG activation), 10 min at 95°C (polymerase activation) followed by 40 cycles of: 15 s at 95°C (denaturation), 30 s at 60°C (annealing) and 30 s at 72°C (extension). To confirm the specificity of amplification signal, gene dissociation curve was analysed in each case. Normalized relative expression levels of target genes in tested samples were calculated using the method described previously by Pfaffl *et al*. [18], based on average Ct value for each sample and average PCR efficiency for each gene. Non-template Control (NTC) reaction was used to identify possible PCR contamination. Diluted mixture of cDNA from all tested samples was used as control to normalize the expression levels of target genes. All experiments were conducted in at least three biological replicates (in the case of NSC and hESCs cells were collected at different time points and passage number).

### Immunocytochemistry

For immunocytochemical analyses monolayer cell cultures were fixed with 4% paraformaldehyde in PBS for 10 min and permeabilized with 0.1% Triton X-100 for 10 minutes at room temperature. Nonspecific binding sites were blocked by incubation with 2% donkey serum (Sigma Aldrich) in PBS for 1 h. For double immunolabeling, fixed cells were subsequently incubated with the appropriate primary antibodies (S2 Table) for 1 h at room temperature. Double labeling was visualized by simultaneous incubation with a combination of species-specific fluorochrome-conjugated secondary antibodies (1 h, room temperature) (S2 Table). Control samples were incubated with the secondary antibodies alone. Slides were mounted with ProLong^®^ Gold Antifade Reagent or ProLong^®^ Gold Antifade Reagent with DAPI (Molecular Probes, Invitrogen, Life Technologies Group), coverslipped and examined using Nikon Eclipse Ci-S epifluorescence microscope.

### BrdU incorporation assay

To assess proliferation ability of NSC, ebiNSc, SiNSc-like and SMiNSc-like cells in fifth passage, 10 μM BrdU was added to NS cell cultures. After 48–120 h of incubation tested cultures were processed for immunocytochemical BrdU co-staining. Firstly, an immunocytochemical staining for SOX2 marker was performed as described, up to the step of PBS washing after incubation with the secondary antibodies (S2 Table). Next, cells were post-fixed in 4% paraformaldehyde and permeabilized with 0.1% Triton X-100 for 10 min at room temperature. Nonspecific binding sites were blocked by incubation with 2% donkey serum in PBS for 30 min. After blocking, cells were treated with 2N HCl in 37°C for 40 min and then with 0.1 M borate buffer (pH 8.5) at room temperature for 12 min. Then, cells were incubated with anti-BrdU antibody for 1 h, washed with PBS and incubated with the appropriate secondary antibody at room temperature for 1 h (S2 Table). Finally, cells were mounted with ProLong^®^ Gold Antifade Reagent, coverslipped and examined using Nikon Eclipse Ci-S epifluorescence microscope. For each analysis 100 nuclei were examined. Proliferation rate was defined as the percentage of BrdU-positive cells compared to all cells in the visual field.

### Senescence-Associated (SA)-β-Galactosidase Staining

SA-β-Gal staining was performed following the protocol by Dimri *et al*. [19]. Cells were washed three times with PBS, fixed with cold 3% paraformaldehyde for 5 min and washed two times with PBS for 5 min. Next, a fresh senescence-associated staining solution (1 mg/ml 5-bromo-4-chloro-3-indolyl β-D-galactopyranoside, X-Gal in dimethylformamide (stock 20 mg/ml)/40 mM citric acid/sodium phosphate, pH 6.0/5 mM potassium ferrocyanide/5 mM potassium ferricyanide/150 mM NaCl/2 mM MgCl2), pre-warmed to 37°C, was added and cells were incubated in 37°C (no CO_2_) for 12 h. After incubation cells were washed two times with PBS for 5 min and photographed using Olympus CKX41 microscope. The percentage of the stained cells was calculated.

### Statistical analysis

All statistical analyses were performed using Mann–Whitney U-test and STATISTICA 10 software (Statsoft, USA).

## Results

### Efficiency of lentiviral transduction

**Fig 1:**
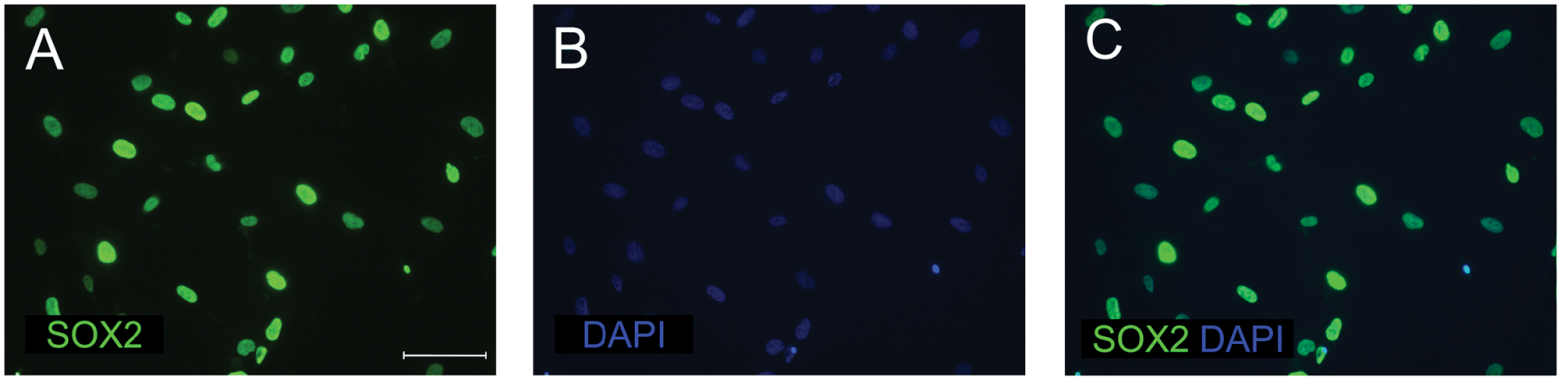
Immunocytochemical detection of SOX2 expression four days after transduction. BJ fibroblasts were stained with anti-SOX2 antibody (A), counterstained with 4’,6-diamidino-2-phenylindole, DAPI (B) and results of staining were merged (C). Images were captured using Eclipse Ci-S epifluorescence microscope with 40x objective; scale bar = 50 μm.

To assess the transduction efficiency, four days after lentiviral infection, immunocytochemical staining for SOX2 expression was performed. All cells were SOX2-positive indicating high transduction efficiency of the lentiviral vector (Fig 1). Nevertheless, after 36 days in culture, substantial repression of SOX2 expression was detected, as only single SOX2-positive cells were observed.

### Reprogramming of BJ fibroblasts into iPSCs

**Fig 2:**
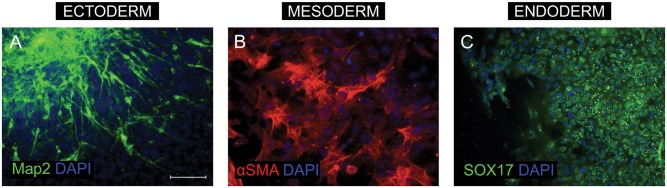
Characterization of induced pluripotent stem cells (iPSCs) derived from BJ fibroblasts by means of lentiviral transduction. iPSCs were spontaneously differentiated *via* embryoid body formation into cells from three germ layers. Differentiated cells expressed markers of ectoderm—MAP2 and presented neuronal morphology (A); mesoderm—alpha-smooth muscle actin (αSMA) and features of cytoskeleton (B); and endoderm—SOX17 (C). Images were captured using Eclipse Ci-S epifluorescence microscope with 20x objective; scale bar = 100 μm.

For this experiment iPSCs were obtained *via* lentiviral transduction of four Yamanaka factors [20,21]. ESC-like colonies were collected, expanded on Geltrex-coated dishes in Essential 8 medium and examined for the expression of pluripotency markers by means of immunocytochemistry. Obtained cells showed expression of SOX2, OCT4, NANOG, Tra-1-60 and Tra-1-81 as well as alkaline phosphatase activity. Additionally, to prove iPSCs identity, results were compared to those for hESCs. Real time PCR analysis showed that SOX2 expression in hESCs and iPSCs was at the same level (hESCs/iPSCs = 1.048; p = 0.8). Moreover, the pluripotent character of these cells was confirmed by their multilineage differentiation potential. iPSCs were spontaneously differentiated into embryoid bodies and examined for expression of markers specific for all three germ layers. Immunocytochemistry showed that resulting cells expressed markers of ectoderm—presence of microtubule associated protein 2 (MAP2) and neuronal morphology; mesoderm—alpha-smooth muscle actin (αSMA) and features of cytoskeleton and endoderm—SOX17 (Fig 2). Detailed expression profile of pluripotency and lineage specific genes (assessed with TaqMan Scorecard kit; Life Technologies) in established induced pluripotent stem cell lines generated in Celther Polska Laboratories was presented by Drozd *et al*. [16].

### Generation and characterization of induced neural cells (ebiNSc and iNSCs-like cells)

**Fig 3:**
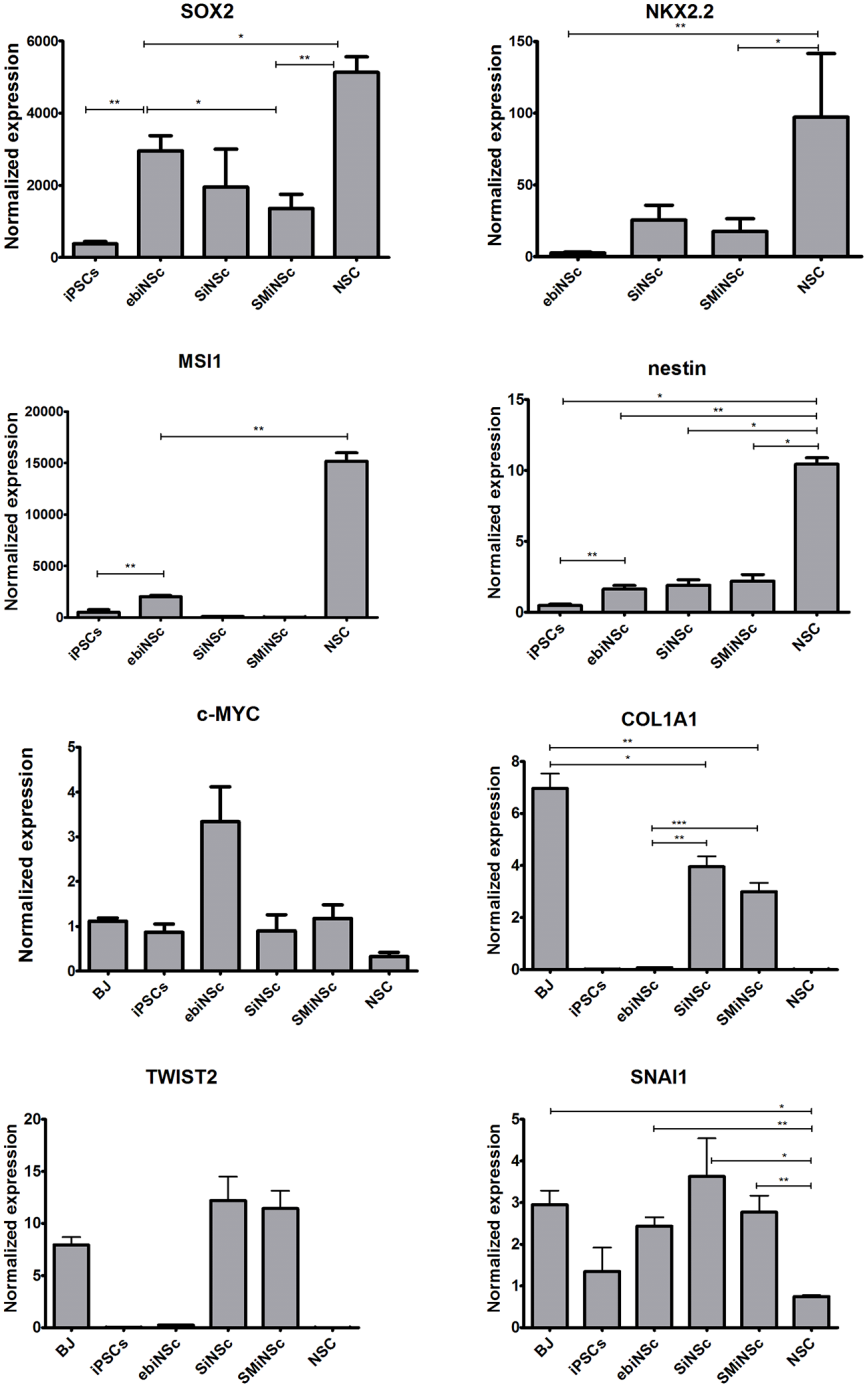
The results of real-time PCR analysis. Expression of each gene in each analyzed sample was firstly normalized to cDNA from all analyzed samples (pooled cDNA) and compared to normalized expression in BJ fibroblasts (SOX2, MSI1, nestin) and iPSCs (NKX2.2). The p-value was indicated in the selected samples (* p < 0.05; ** p < 0.01; *** p < 0.001; SEM), all statistical data are summarized in S4 Table.

Real-time PCR analysis was performed for all obtained types of iNc to investigate the expression levels of markers characteristic for neural stem cells (SOX2, nestin, Musashi-1 –MSI1 and NKX2.2), markers of mesenchymal transition (COL1A1, SNAI1, TWIST2) and c-MYC. As a positive control H9-hESC-derived NSC cell line was used. Results were also compared to starting population of cells: iPSCs and BJ fibroblasts. All results are presented as graphs in linear scale. For each sample expression values were normalized to pooled cDNA from all tested samples and compared to BJ fibroblasts (*SOX2*, *MSI1*, *nestin*, *c-MYC*) and iPSCs (*NKX2*.*2*) in Fig 3 and S3 Table.

The highest expression of neural stem cells-associated genes was observed in NSC. Nevertheless, an elevated level of SOX2 was also detected in ebiNSc and directly reprogrammed SiNSc-like and SMiNSc-like cells (2955.8, p = 0.008; 1953.2 p = 0.014; 1360.5 p = 0.006, fold respectively). Although the expression level of nestin was low in negative controls—BJ fibroblasts and iPSCs, it was still found to be 10.43 (p = 0.03) and 21.66 (p = 0.03) times lower when compared to NSC, respectively. In the remaining cells the expression level of nestin was at a similar level: SMiNSc-like cells (2.2, p = 0.11), SiNSc-like cells (1.9, p = 0.39), ebiNSc (1.64, p = 0.27). Such small discrepancies between fibroblasts (negative control) and induced neural cells result from the fact that fibroblasts express nestin [22–25]. Expression of other neural stem cell markers (MSI1, NKX2.2) was detected mainly in NSC. The highest level of NKX2.2 was observed in SiNSc-like cells, while the lowest in ebiNSc (NSC/SiNSc-like = 5.7, p = 0.07; NSC/SMiNSc-like = 9.23, p = 0.028; NSC/ebiNSc = 66.38, p = 0.008; ebiNSc/iPSCs = 1.47, p = 0.8); however, these differences among induced neural cells were statistically insignificant. Considering MSI1, directly reprogrammed cells did not show expression of this gene; however, its level in ebiNSc was 2016.6 (p = 0.008) times higher than in the negative control. None of these genes was expressed in BJ fibroblasts. Concerning the influence on direct reprogramming, c-MYC expression was analyzed after 3–7 weeks following transduction. These results show that ebiNSc, not SMiNSc-like cells, as expected, exhibit the highest level of this gene. In respect to BJ fibroblast c-MYC expression value was 2.9 (p = 0.23) for ebiNSc, 1.06 (p = 0.86) for SMiNS-like cells, 0.81 (p = 0.7) for SiNS-like cells and 0.23 (p = 0.052) for NSC.

Expression of mesenchymal marker genes (*COL1A1*, *TWIST2*, *SNAI1*) was detected mainly in BJ fibroblasts and directly reprogrammed cells. Expression of TWIST2 in iNSCs-like cells was even higher than in the initial cell line—BJ fibroblasts (SiNSc-like/BJ = 1.53, p = 0.19; SMiNSc-like/BJ = 1.44, p = 0.15). When compared to fibroblasts, the expression of *COL1A1* was significantly lower in SiNSc-like cells (BJ/SiNSc-like = 1.76, p = 0.014) and SMiNSc-like cells (BJ/SMiNSc-like = 2.33, p = 0.0058). Trace levels of *TWIST2* and *COL1A1* expression were found in ebiNSc (BJ/ebiNSc = 33.48, p = 0.008 and 87.51, p = 0.008, respectively). Interestingly, expression of SNAI1 was detected in all analyzed samples (BJ/iPSCs = 2.19, p = 0.11; BJ/ebiNSc = 1.21, p = 0.27; BJ/NSC = 3.97, p = 0.03), reaching the highest level in SiNSc-like cells (SiNSc-like/BJ = 1.23, p = 0.88) and SMiNSc-like cells (BJ/SMiNSc-like = 1.07, p = 0.93).

**Fig 4:**
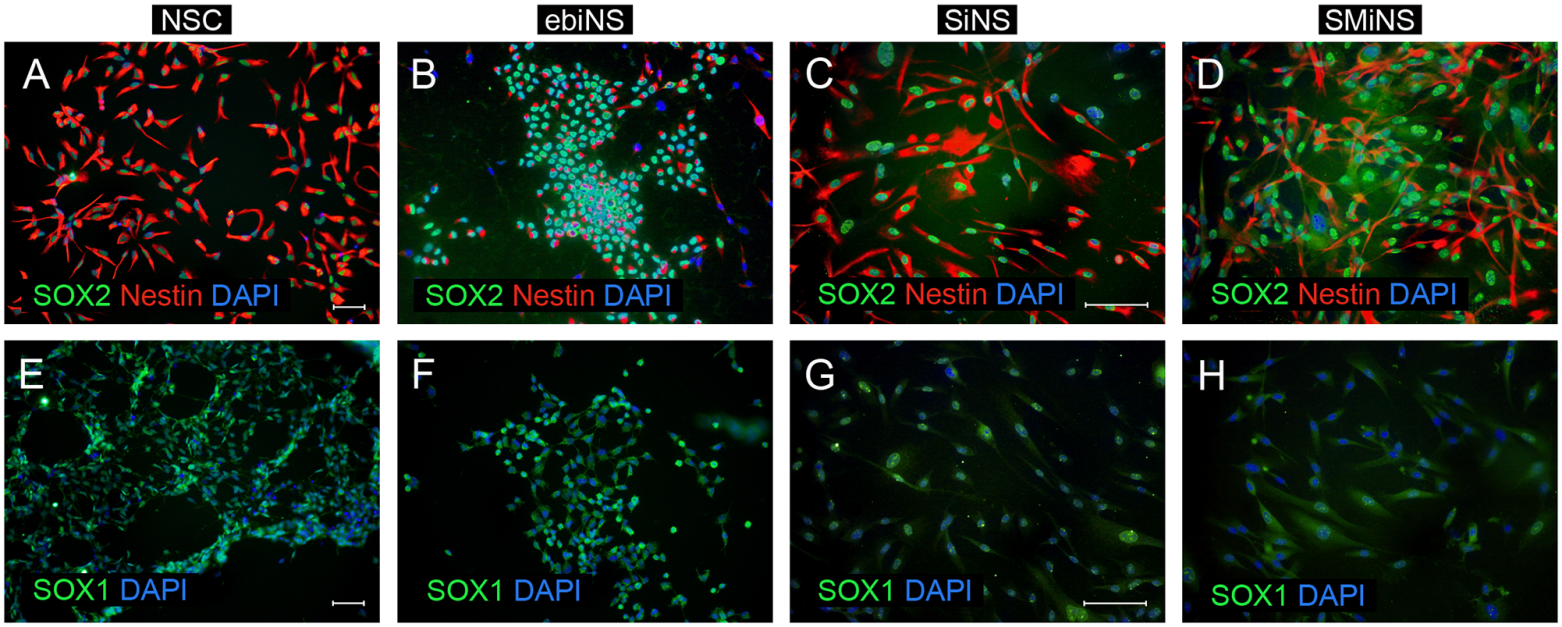
The expression of three neural stem cell markers SOX2, nestin and SOX1 in NSC (A, E), ebiNSc (B, F), SiNSc-like cells (C, G) and SMiNSc-like cells (D, H). Images were captured using Eclipse Ci-S epifluorescence microscope with 20x (A, B, E, F) and 40x objective (C, D, G, H); scale bar = 50 μm.

In order to determine the identity of obtained cells, immunocytochemical staining was performed two weeks after culture establishment (Fig 4). In contrary to NSC cell line, characterized by homogeneous expression of SOX2, SOX1 and nestin, all types of iNc were slightly heterogeneous. Despite the fact that the vast majority of cells showed co-expression of analyzed neural stem cell markers (SOX2, SOX1 and nestin), some cells were only SOX2-positive, nestin-positive or did not express any of these markers. Moreover, the morphology of ebiNSc resembled that of NSC (small cells, with bipolar morphology), whereas directly reprogrammed cells were characterized by more elongated spindle, in most cases resembling mesenchymal cells. Further analysis revealed that ebiNSc showed point expression of integrin-beta-1 in the cytoplasm and nuclear expression of MSI1 (data not shown). As a negative control, BJ fibroblasts were used. No expression of SOX1 and SOX2 was detected, however, single cells were positively stained for nestin.

### The effectiveness of ebiNSc, SiNSc-like and SMiNSc-like cells generation

**Fig 5:**
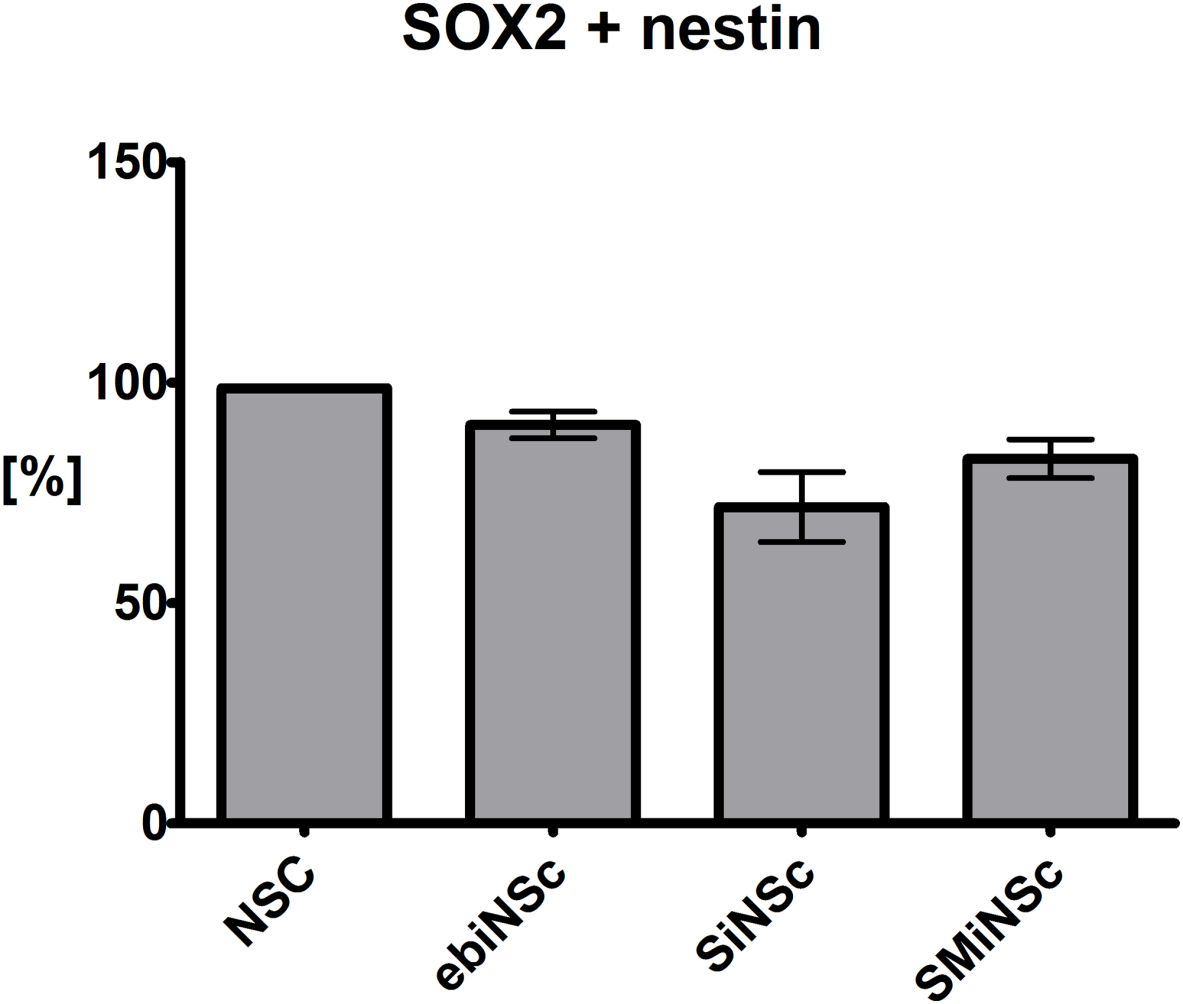
The effectiveness of induced neural stem cell generation presented as the percentage of NSC, ebiNSc, SiNSc-like cells and SMiNSc-like cells expressing simultaneously SOX2 and nestin (SEM).

The procedure of differentiating iPSCs to induced neural stem cells consisted of four distinct steps, within which the morphology of cells has been changing substantially. Starting from iPS colonies, cells went through embryoid bodies stage (cultured with retinoic acid and neural induction medium), ending in monolayer of differentiated cells with NSC-characteristic morphology. The identity of generated cells was confirmed by immunocytochemical staining. Almost all NSC cells (98.69 ± 0.35%) were SOX2/nestin double positive at the second passage (Fig 5). Alike the NSC cells, nearly all ebiNSc expressed NSC markers, including SOX2, SOX1, nestin, MSI1 and vimentin. In case of ebiNS cells, the portion of SOX2/nestin double positive cells reached 90.36 ± 3.01% (Fig 5).

SOX2 and SOX2-MYC-directly reprogrammed cells, cultured in medium dedicated for neural stem cell culture, began to slowly disperse and to form star-shaped interconnected clusters. Following the incubation in suspension culture for at least 7 days and subsequent re-transferring of cells into monolayer conditions, these cells began to resemble neural stem cells. Importantly, not all attempts of direct reprogramming led to successful production of iNSCs-like cells. Some attempts yielded no SOX2- or SOX1-positive cells. On the other hand, in experiments considered as propitious, the potency of iNSCs-like cells generation yielded 71.76 ± 7.92% and 82.69 ± 4.39% for SiNSc-like and SMiNSc-like cells, respectively (Fig 5). Importantly, not every SOX2/nestin-positive cell acquired neural morphology. It should be noted that the number of successful attempts was higher in the case of SMiNSc-like than SiNSc-like cells (4/5 and 3/8).

### Multipotency of ebiNSc, SiNSc-like and SMiNSc-like cells

**Fig 6:**
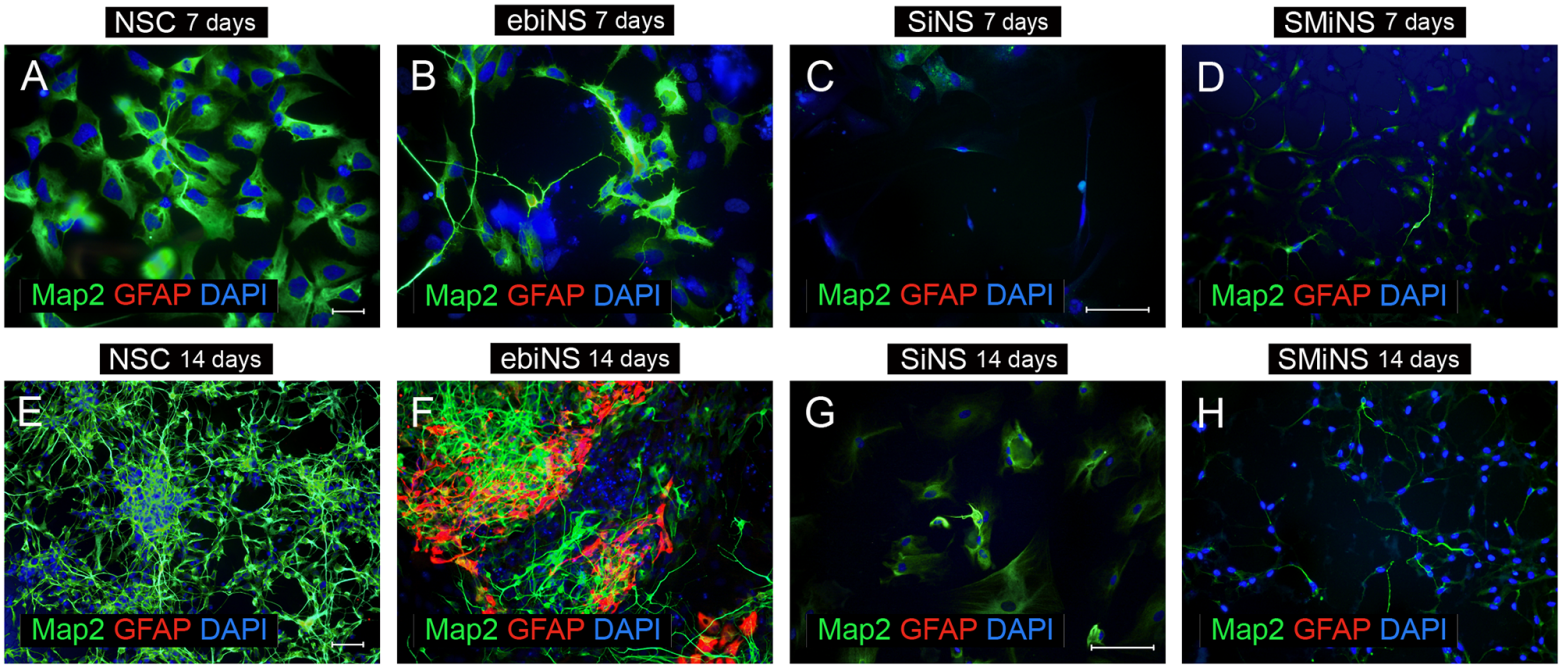
The expression of MAP2 and GFAP in NSC (A, E), ebiNSc (B, F), SiNSc-like cells (C, G) and SMiNSc-like cells (D, H) after 7 and 14 days of differentiation in the neural induction medium. Images were captured using Eclipse Ci-S epifluorescence microscope with 20x (A, B, E, F) and 40x objective (C, D, G, H); scale bar = 50 μm.

**Fig 7:**
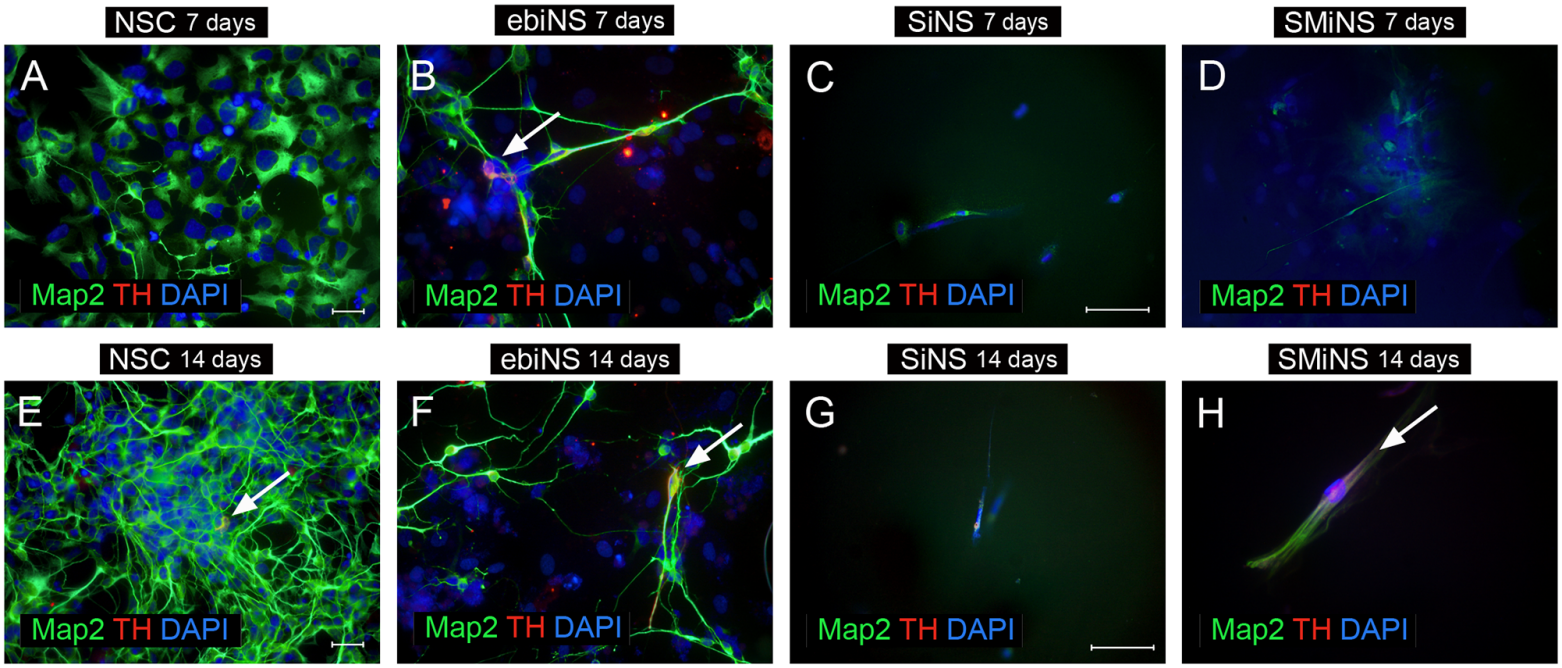
The expression of MAP2 and TH in NSC (A, E), ebiNSc (B, F), SiNSc-like cells (C, G) and SMiNSc-like cells (D, H) after 7 and 14 days of differentiation in the neural induction medium. Images were captured using Eclipse Ci-S epifluorescence microscope with 20x (A, B, E, F) and 40x objective (C, D, G, H); scale bar = 50 μm.

Upon 7 days of differentiation, almost all types of neural cells presented MAP2 expression. Although the morphology of differentiated NSC cells (positive control) was mostly homogeneous (large, flattened cells), single cells resembling neurons (with clearly marked soma, axons and dendrites) with more intense MAP2-positive signal (Fig 6). Within these cells, neither tyrosine hydroxylase (TH)-positive nor GFAP-positive cells were detected (Figs 6 and 7). Culturing NSC for 14 days in differentiation medium did not result in any GFAP-positive cell, but single TH-expressing cells were observed. Moreover, cells substantially changed their morphology and the majority of cells, with long axons and dendrites, began to form clusters arranging dense networks.

**Fig 8:**
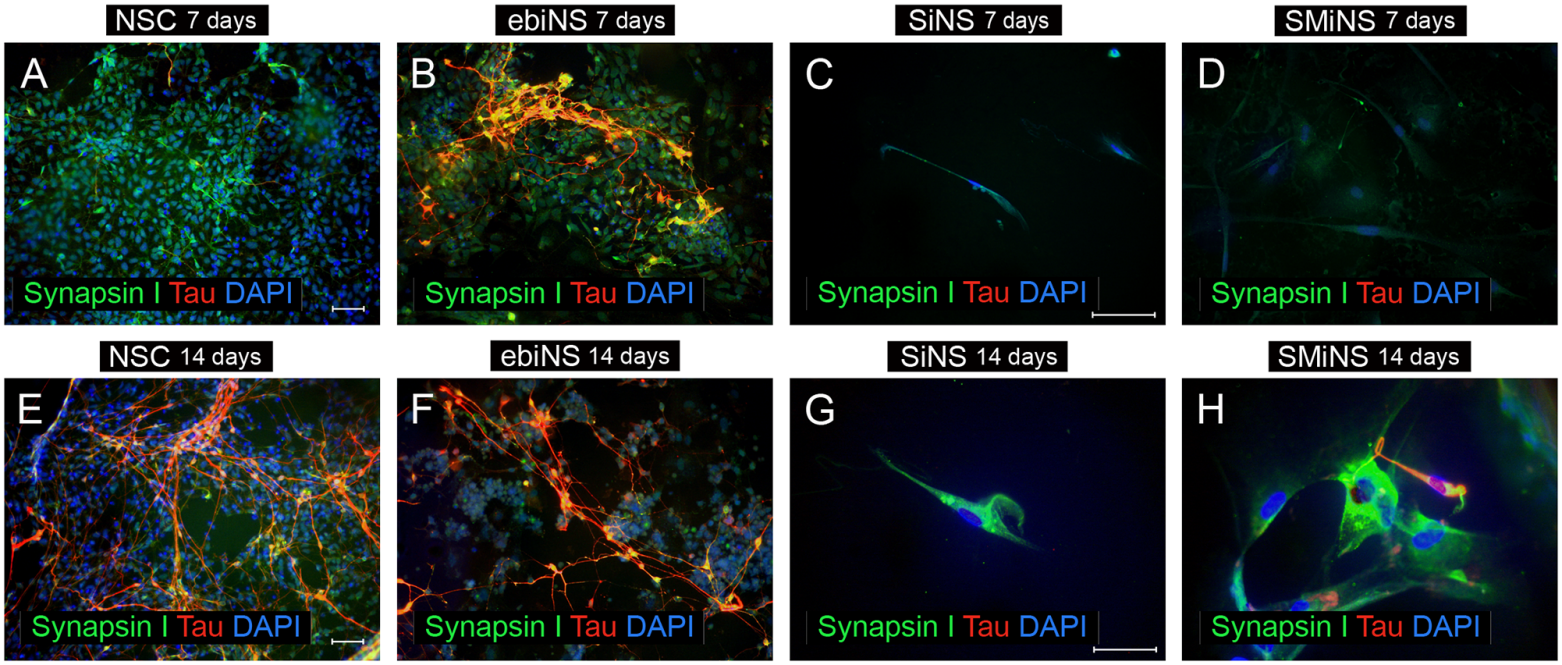
The expression of Synapsin I and Tau in NSC (A, E), ebiNSc (B, F), SiNSc-like cells (C, G) and SMiNSc-like cells (D, H) after 7 and 14 days of differentiation in the neural induction medium. Images were captured using Eclipse Ci-S epifluorescence microscope with 20x (A, B, E, F) and 40x objective (C, D, G, H); scale bar = 50 μm.

**Fig 9:**
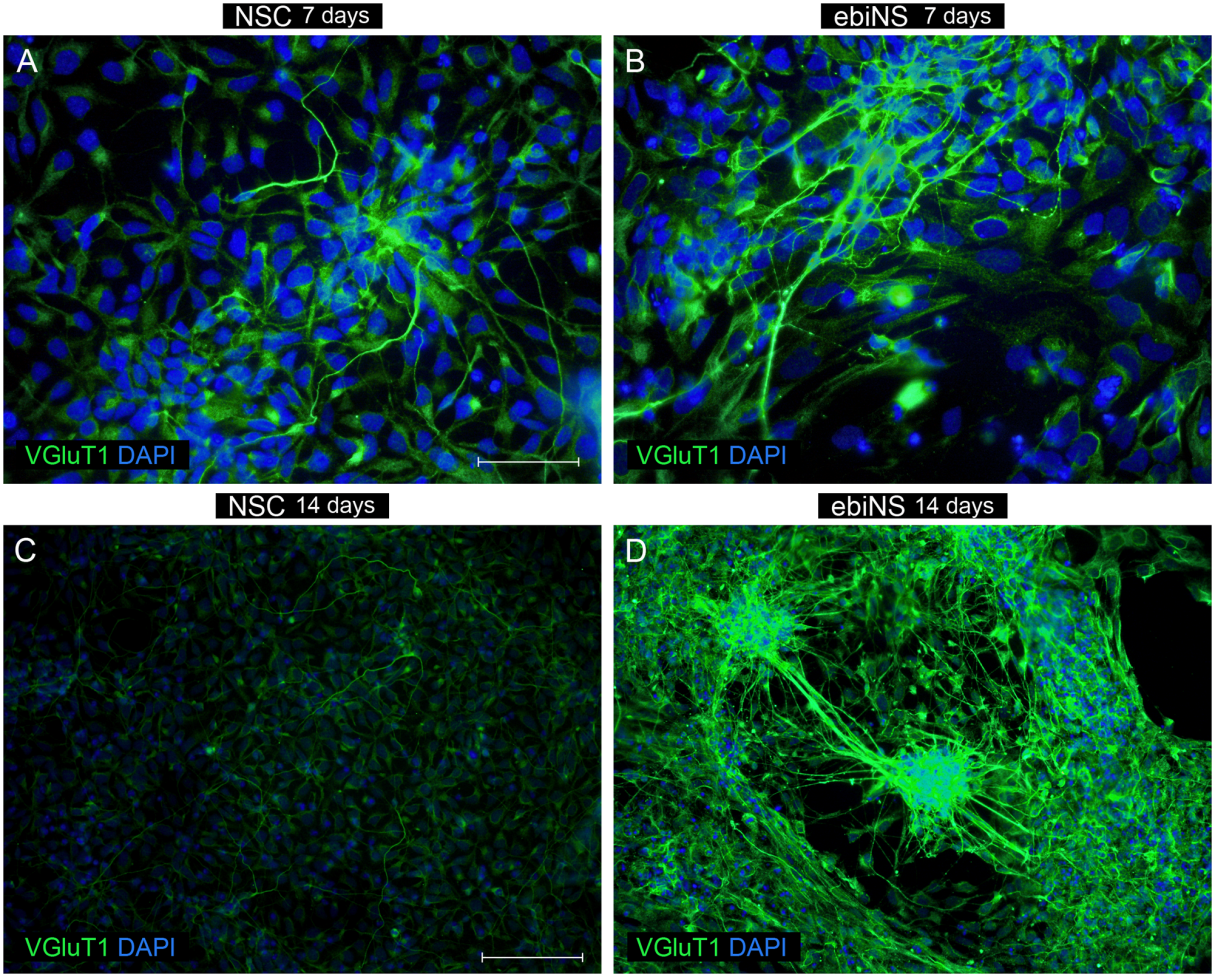
The expression of VGLUT1 in NSC (A, C) and ebiNSc (B, D) lines after 7 and 14 days of differentiation in the neural induction medium. Images were captured using Eclipse Ci-S epifluorescence microscope with 20x objective; scale bar = 100 μm.

The number of ebiNSc-derived neuronal cells at 7^th^ day of differentiation was significantly lower comparing to NSC. Nevertheless, ebiNSc-derived neuronal cells had more mature and distinct morphology—ranging from MAP2-positive flattened cells with short fibers to uni-, bi- and multipolar neuronal cells (Fig 6). During following 7 days of differentiation, this morphology did not significantly change. Additionally, after 14 days in these conditions, single GFAP-positive cells were detected (Fig 6). Single astrocytic cells were observed as early as on day 9 of differentiation. Surprisingly, at early time points, most GFAP-positive cells co-expressed MAP2. The total number of TH-positive cells obtained from ebiNSc was higher comparing to cells obtained from NSC. After a week of differentiation, hundreds of TH-positive cells among ebiNSc and single TH-positive cells among NSC were detected (Fig 7). The difference was also observed in Tau expression level—Tau-positive cells appeared after one week of differentiation in both NSC and ebiNSc cell lines (Fig 8), but again the number for ebiNSc-derived cells was higher (varied from experiment to experiment, but at least 1.4-times higher). Tau-positive cells additionally presented an enhanced expression of Synapsin I. After two weeks, the number of cells expressing Tau and Synapsin I increased in both NSC and ebiNSc cultures. After one week of culture in neural induction medium, the NSC differentiated into single VGLUT1-positive cells with intense signal (Fig 9). The majority of these cells showed flattened morphology with point expression of this protein in the cytoplasm. Within ebiNSc culture, three populations of cells were identified—VGLUT1-positive cells with typical neuronal morphology (the number of these cells was higher in comparison to cells differentiated from NSCs), flattened cells with VGLUT1 expression localized around the nucleus and undifferentiated stromal cells with no expression of this marker (Fig 9).

Moreover, neuronal cells derived after 7 days of ebiNSc differentiation were subjected to real-time PCR analysis to examine the level of GAD65 expression. GAD65, one of two isoforms of glutamic acid decarboxylase enzyme, is involved in decarboxylation of glutamate to CO_2_ and γ-aminobutyric acid (GABA)–the major inhibitory neurotransmitter in the mammalian brain [26]. GAD65 is localized to nerve terminals and synthesizes GABA specifically for neurotransmission [27]. Real-time PCR analysis showed that expression of GAD65 in ebiNSc-derived neuronal cells after 7 days of differentiation increased 591.7 ± 45.26 when compared to BJ fibroblasts and 34.92 ± 0.89 when compared to undifferentiated ebiNSc. This result strongly suggest that ebiNSc differentiation leads to the formation of functional neuronal cells that are capable of neurotransmitters synthesis.

Fourteen days of differentiation of SiNSc-like cells resulted in single MAP2-positive cells, but in the majority of these cells signal was weak and non-specific, since morphology was rather mesenchymal than neuronal (Fig 6). At the same time, SMiNSc-like cells were able to differentiate into single bipolar neuronal cells (Fig 6). In both cases, no astrocytic cells were obtained. iNSCs-like cells differentiated into single TH-positive cells (Fig 7). After 14 days of differentiation of SiNSc-like cells, single Synapsin I-positive cells were present but no Tau-positive cells were observed (Fig 8). On the other hand, SMiNSc-like cells differentiated into single Synapsin I-positive cells and single Tau/Synapsin I double positive cells, but some of these cells did not present typical neuronal morphology (Fig 8).

### The potency of ebiNSc, SiNSc-like and SMiNSc-like cells differentiation into neuronal cells

**Fig 10:**
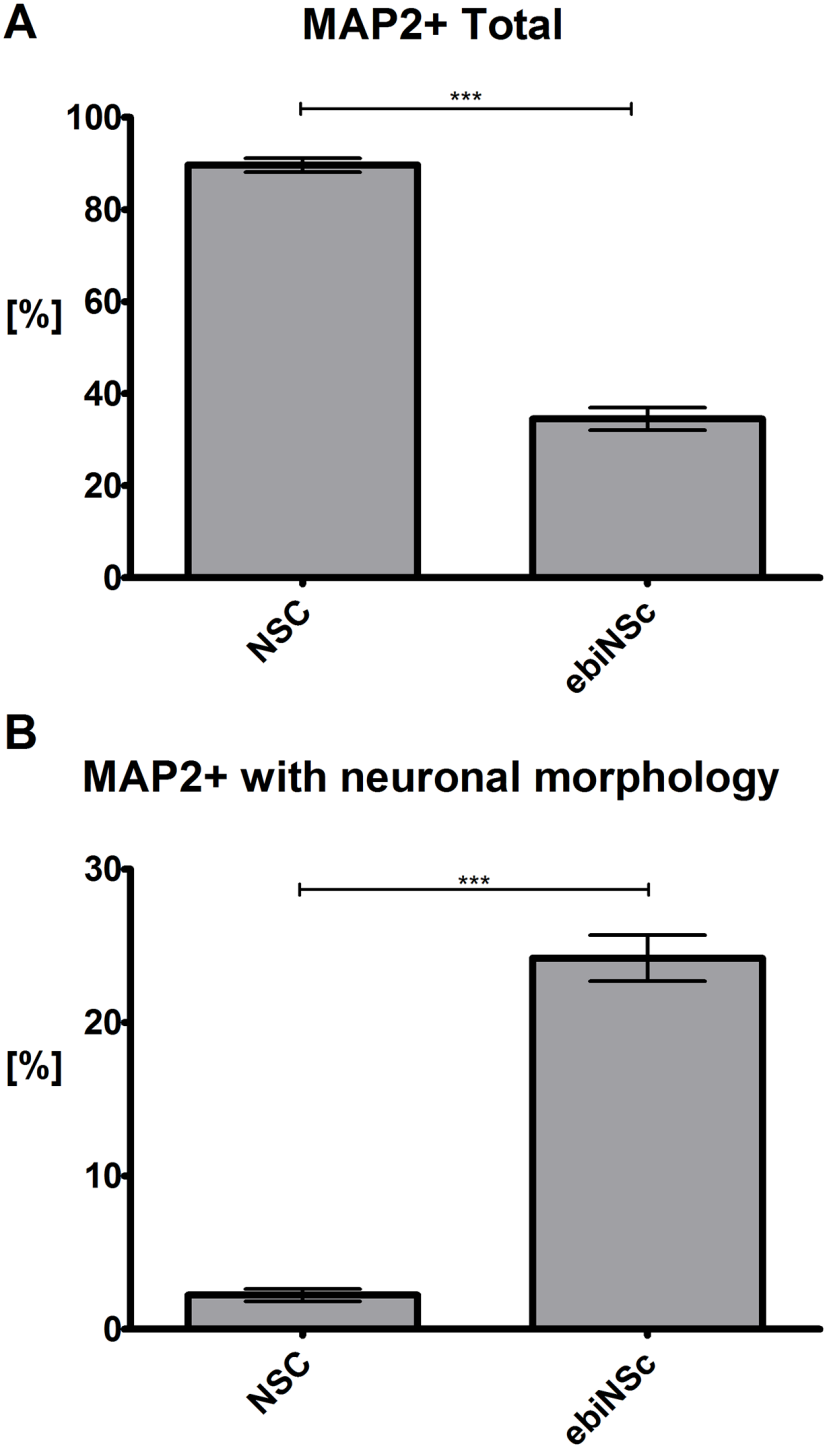
The potency of neuronal cells generation. Comparing the total number of MAP2-positive cells (A) and MAP2 expressing cells with distinctive neuronal morphology (B) within the population of NSC and ebiNSc after seven days of differentiation (SEM).

NSC presented the highest total potency of neural differentiation, reaching 89.63 *±* 1.52% of MAP2-positive cells after 7 days of differentiation (Fig 10A). On the 7^th^ day, the number of MAP2-positive cells differentiated from ebiNSc was significantly lower compared to NSC– 34.51 *±* 2.44% of the total population (NSC/ebiNSc = 2.60, p<0.001) (Fig 10A). Nevertheless, most of these cells presented a typical neuronal morphology (24.19 ± 1.5%), whereas only 2.22 ± 0.40% of MAP2-positive NSC-derived cells were elongated (ebiNSc/NSC = 10.89, p<0.001) (Fig 10B). After 14 days of differentiation, both ebiNSc and NSC formed dense neuronal networks and thus could not be counted. In case of directly reprogrammed cells differentiation, only single MAP2-positive cells were present in the culture. However, comparing SMiNSc-like to SiNSc-like cells, more MAP2-positive cells were observed in the former type. Interestingly, out of all analyzed iNc, astrocytic cells were obtained only from ebiNSc following the two-week differentiation period.

### BrdU incorporation assay and Senescence Associated (SA)-β-Galactosidase Staining

**Fig 11:**
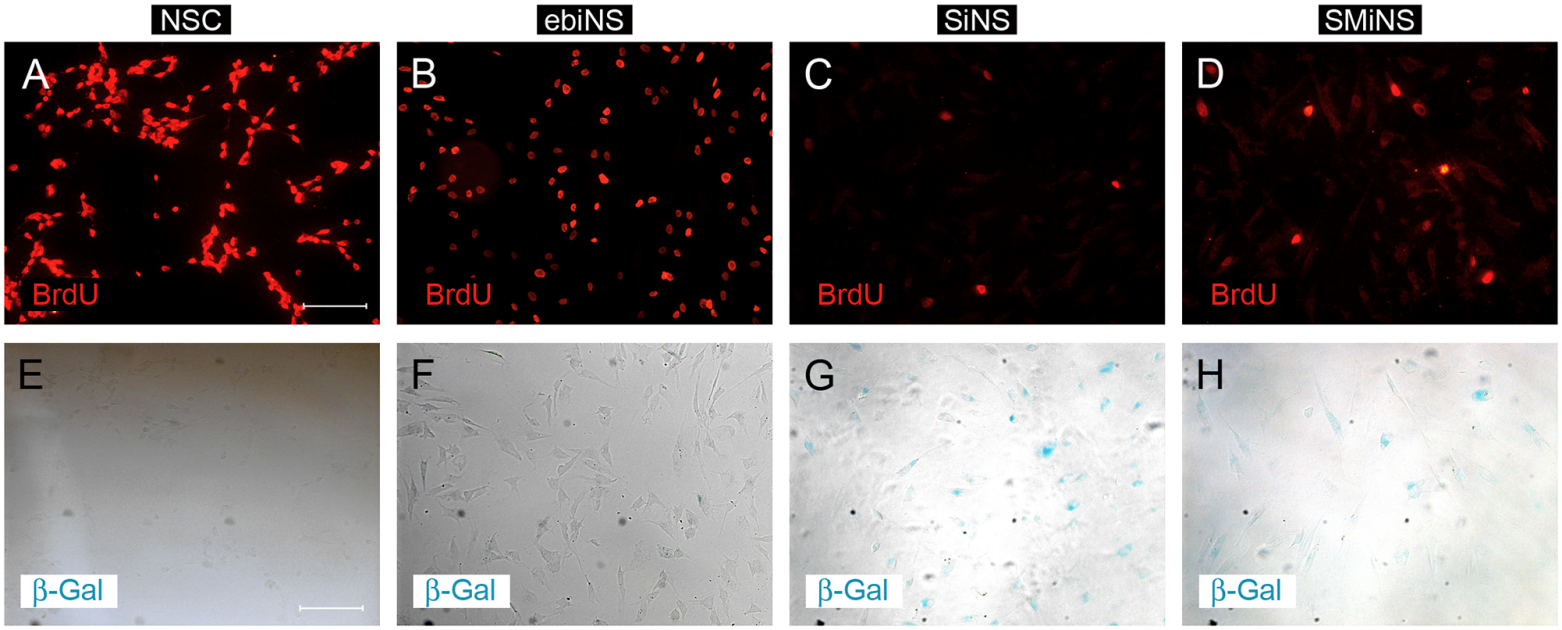
The results of BrdU incorporation assay and Senescence-Associated (SA)-β-galactosidase staining obtained for NSC (A, E), ebiNSc (B, F), SiNSc-like cells (C, G) and SMiNSc-like cells (D, H) after 48 h of incubation. Images were captured using Eclipse Ci-S epifluorescence microscope with 20x objective; scale bar = 100 μm.

**Fig 12:**
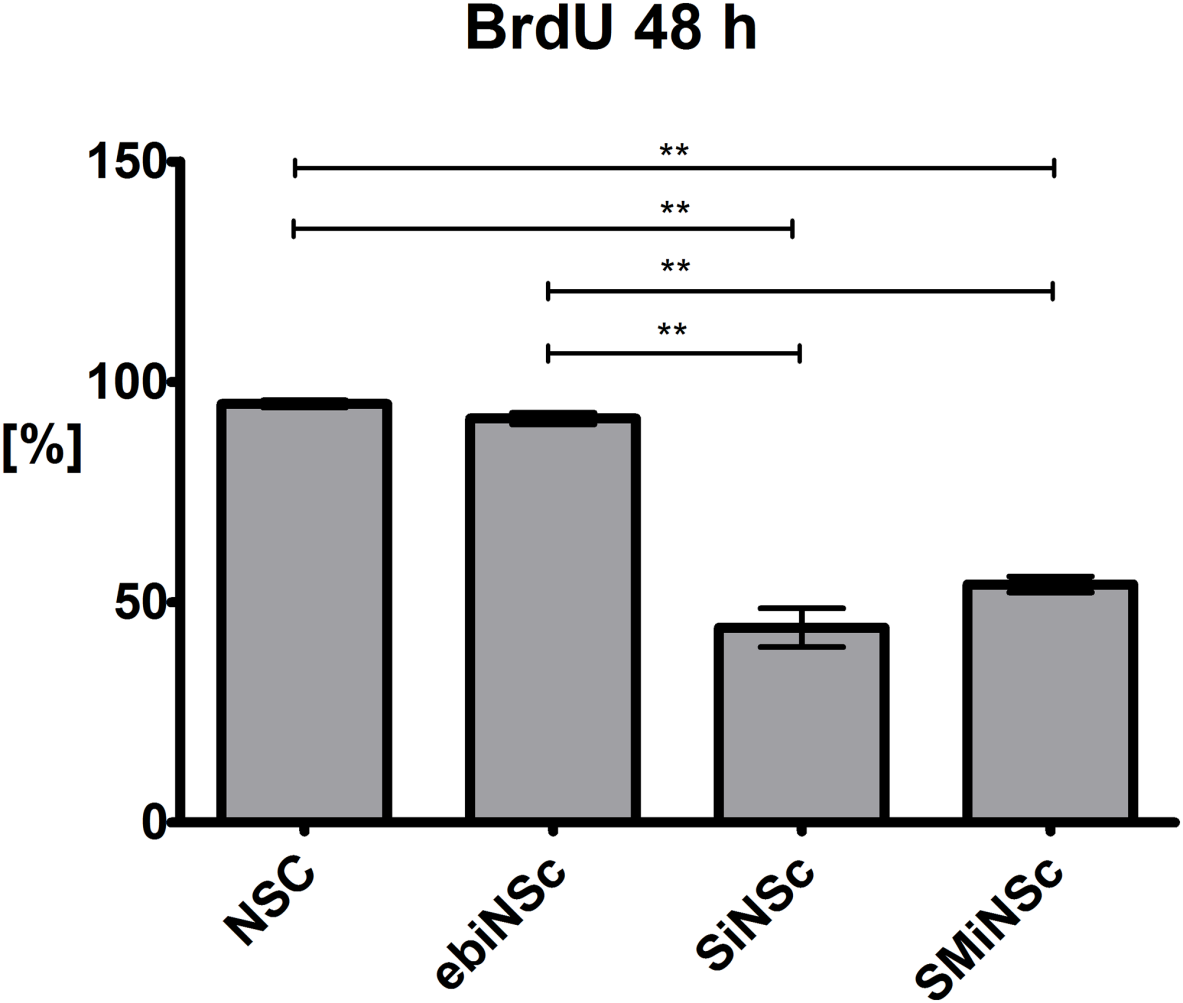
Incorporation of BrdU in analyzed cell lines after 48 h of incubation (SEM).

Forty-eight hours after incubation with BrdU, significant differences in the proliferation level of ebiNSc in comparison to directly reprogrammed cells were revealed (Fig 11). The highest number of BrdU-positive cells was observed in NSC (94.99 ± 0.81%) and ebiNSc (91.68% ± 1.28%) while in the case of SMiNSc-like and SiNSc-like cells, BrdU was incorporated in only 54.05 ± 1.81% and 44.2 ± 4.38% of cells, respectively (Fig 12). After prolonged incubation (120 h) no increase in the proliferation rate of SiNSc-like and SMiNSc-like cells was reported. Actually the number of BrdU-positive cells was even lower (27.86 ± 7.86% and 42.92 ± 3.07%, respectively), most probably due to the fact that non-proliferating cells started to detach from the surface of culture plates. Moreover, extended incubation time did not influence the proliferation rate of NSC or ebiNSc. Senescence-Associated (SA)-β-galactosidase staining performed after 48 h of culture revealed that the majority of directly reprogrammed iNSCs-like cells underwent senescence, whereas only single SA-β-Gal-positive cells were observed in case of NSC and ebiNSc.

## Discussion

Two methods of induced neural stem cells generation from somatic cells have been described so far: direct reprogramming with combination of specific factors as well as iPSCs generation and further differentiation. Hereby we present the first systematic comparison, regarding gene expression profile, differentiation potential and proliferation properties, of induced neural cells obtained *via* different methods. Direct reprogramming was performed by lentiviral transduction of either SOX2 or SOX2 with c-MYC. Surprisingly, not all experiments were considered as propitious (not resulting in generation of SOX1-, SOX2- and nestin-positive cells). Introduction of a second reprogramming factor—c-MYC, positively affected the repeatability of the experiment. Furthermore, the obtained iNSCs-like cells were more susceptible to stabilization. It is possible that c-MYC enhances cells vulnerability to changes, resulting not only in the increased yield, but also in elevated susceptibility of cells to reprogramming. Thereby this factor increases reproducibility of this process. c-MYC interacts with histone methyltransferases, which leads to changes in chromatin structure, and thus elevates efficiency of reprogramming into iPSCs [28]. c-MYC appears to be a key factor responsible for so called “first transcriptional wave”, which is characterized by high rate of cell proliferation, changes in metabolism, cytoskeletal organization and down-regulation of genes associated with development and mesenchymal-to-epithelial transition [29]. Moreover, the Myc family genes play an important role in neurogenesis, as they are expressed during fetal development of brain and c-MYC is involved in maintenance of homeostasis in neural stem cells [30,31] *via* various signaling pathways (e.g. Miz-1 or CIP2A) [30,31]. Real-time PCR analysis after 3–7 weeks post-transduction revealed no significant differences in c-MYC mRNA level between SiNSc-like and SMiNSc-like cells. This suggests that c-MYC played a key role at the beginning of reprogramming and its level decreased over the time of culture.

SiNSc-like and SMiNSc-like cells showed increased level of NKX2.2 and MSI1. Moreover, SOX2 expression in these cells was even 1953.2 (p = 0.014) and 1360.5 (p = 0.006) times higher, respectively, when compared to BJ fibroblasts, from which they were generated. Poor differentiation results, simultaneous expression of neural stem cell and mesenchymal markers and lack of long-term survival ability suggest that SiNSc-like and SMiNSc-like cells were not fully reprogramed. Lentiviral transduction efficiency analysis conducted at the beginning of direct reprogramming (Fig 1) points to repression of SOX2 expression during the culture as the partial cause of differences in gene expression profile and differentiation properties between ebiNSc and directly reprogrammed cells. Despite application of the protocol by Ring *et al*. [7], the high level of SOX2 expression was not enough to generate fully reprogrammed iNSCs, probably due to some unknown mechanism of SOX2 expression gradual loss. Techniques used in this study were adequate and properly conducted, since we are able to efficiently obtain induced pluripotent stem cells from different types of somatic cells with different techniques and maintain their pluripotent character for many passages, as described by Drozd *et al*. [16].

Lower initial number of SOX2/nestin double positive cells in directly reprogrammed cells, when compared to ebiNSc should not be considered as an issue since these cells are able to proliferate and thus can be further expanded. However, SiNSc-like and SMiNSc-like cells were gradually losing their proliferative potential with every passage, as confirmed with BrdU incorporation assays (Figs 11 and 12). The proliferation assay results for directly reprogrammed cells are contradictive to ebiNSc, which can be cultured for more than 15 passages without any loss of proliferative potential and multipotency. Moreover, after the 3^rd^-5^th^ passage, depending on the experiment, directly reprogrammed cells either began to detach or became senescent (Fig 12). Different conditions of SiNSc-like and SMiNSc-like cells culture were tested in independent experiments. Despite protocols modifications (e.g. different culture media—ReNcell, StemPro NSC SFM, or different plate coating—Geltrex, Poly-L-Ornithine/Laminin) senescence in these cells was inevitable. Senescence is considered as one of the factors limiting the efficiency of reprogramming cells into iPSCs. The reprogramming process, by changing cell fate with introduction of transgenes, is inextricably linked with cellular stress response and further increased expression of p53, p16INK4a, and p21CIP [32]. As the up-regulated expression of senescence effectors was found to be specific for pre-iPSCs [32], we postulate that this phenomenon may also be associated with direct reprogramming, and introduction of even one transcription factor can trigger a cascade of events leading to senescence. It is documented that senescence is a limiting factor in direct conversion of somatic cells to neurons [33]. Herein presented results indicate that direct reprogramming may also be impaired by senescence. In fact, insertion of reprogramming factors causes changes in morphology, gene expression profile of SiNSc-like and SMiNSc-like cells, which partially acquire potential of neural cells, but these process is compromised by proliferation repression. Finally, senescence may be the cause of incomplete reprogramming of iNSCs-like cells. The higher number of successful reprogramming attempts for SMiNSc-like than SiNSc-like cells may indicate that c-MYC at some extent helps to overcome senescence. On the other hand, it cannot be excluded that c-MYC, as an oncogene, may enhance this process—oncogene induced senescence [34]. Therefore, a thorough analysis is essential to determine the mechanism underlying the observed phenomenon. Identification and minimization influence of senescence-promoting factors could increase direct reprogramming efficiency.

The main differences between the methods of direct and indirect iNc derivation are temporal kinetics and transcription factors used. During fibroblast-derived iPSCs differentiation into iNSCs, which lasts about two weeks and consists of four different stages, the morphology of cells changes substantially. Of note, time and efficiency of reprogramming fibroblasts to iPSCs should also be considered in time required for ebiNSc derivation. Depending on the vector used, efficiency of fibroblasts reprogramming varies considerably [21,35].

Contrary to directly reprogrammed cells, ebiNSc (iPSCs-derived induced neural stem cells) presented gene expression profile, differentiation ability and proliferation properties, which were the most similar to NSC. When compared to BJ fibroblasts, ebiNSc showed high expression of SOX2 (2955.8, p = 0.008 fold) and MSI1 (2016.6, p = 0.008 fold) and low expression of mesenchymal markers. It was impossible to analyze NKX2.2 level, since BJ cells did not show any expression of this gene. Surprisingly, the level of nestin was subtly higher in ebiNSc (alike in iNSCs-like cells) than in fibroblasts, as fibroblasts also produce this protein [22–25]. In contrast to NSC, ebiNSc differentiation resulted in a very heterogeneous population with three types of cells—MAP2-positive cells with flat morphology, elongated MAP2-positive cells with neuronal morphology able to form networks, as well as MAP2-negative partially differentiated cells with fibroblast-like morphology. The latter were observed to form stromal cell layer surrounding ebiNSc-derived neuronal cells. This is particularly important since stromal cells were reported to support differentiation [36,37]. Despite the efficiency of neural differentiation of ebiNSc was lower than in case of NSC (34.51 ± 2.44% and 89.63 ± 1.52%, respectively), the ebiNSc-derived cells were morphologically and physiologically more mature than NSC-derived neuronal cells after 7 days of differentiation. After another 7 days of differentiation, the number of cells expressing markers of mature (Tau) and functional (TH—dopaminergic, VGLUT1 —glutamatergic) neuronal cells was higher within ebiNSc-derived neuronal cells than within NSC-derived population. Real time PCR analysis confirmed that the resulting neuronal cells are capable of GAD65 synthesis, which is in turn responsible for synthesis of GABA—the major inhibitory neurotransmitter in the mammalian brain. Furthermore, GFAP-positive cells were observed only within ebiNSc-derived population. Surprisingly, at early stage of differentiation, most GFAP-positive cells were also positive for MAP2, what is a normal stage of astroglial development [38]. Finally, GFAP-positive astrocytic cells identified among differentiated ebiNSc could support neuronal differentiation of these cells [39–41].

When compared to BJ, ebiNSc presented higher level of key genes expression, but still it was several times lower than in NSC. The discrepancies between ebiNSc and NSC may suggest that ebiNSc could be not fully developed or represent some immature type of neural stem cells. Nevertheless, this hypothesis was not confirmed by any multipotency analyses. The differences between NSC and ebiNSc may be caused by the origin of these cells –NSCs were derived from hESCs, while ebiNSc originated from iPSCs. Many publications deliberate on subtle differences in expression levels of several genes [42–44], mRNAs [44] and microRNAs [45], between human ESCs and iPSCs. Additionally, when analyzing iPSCs, their gene expression [46], genetic background [47] and “epigenetic memory” [48] should also be considered. It has also been reported that iPSCs show line-to-line variability [49] what may influence their differentiation potency [50,51]. These differences may be especially considered when analyzing cells of iPSCs and hESCs origin [52].

Prior to introduction of NSC derivation techniques from embryonic stem cells or their generation from induced pluripotent stem cells, these cells (*e*.*g*. NSCs from biopsies) were characterized by limited proliferative potential under *in vitro* culture conditions. So called normal human astrocytes (NHA, Lonza, Switzerland) constitute a good example of GFAP-positive neural stem cells which may be cultured for only a few passages before they become senescent [53,54]. There are two possible explanations for such differences. Firstly, cells such as NHA or directly reprogrammed iNSCs-like cells are in fact neural progenitors (non-multipotent stem cells), thus they cannot proliferate indefinitely like multipotent cells derived from ESCs. Alternatively, it is possible to directly generate neural multipotent stem cells but the *in vitro* cell culture conditions do not resemble their *in vivo* niche and therefore do not promote self-renewal of these cells. The second hypothesis is based on the assumption that neural stem cells derived from ESCs or iPSCs self-renew themselves more easily under *in vitro* conditions than directly reprogrammed iNSCs. Similarly, difficulties are associated with maintenance of hematopoietic stem cells self-renewal potential under *in vitro* culture conditions [28].

Since normal human astrocytes (GFAP-positive neural stem cells or neural progenitors) has been characterized as capable of glial and neuronal differentiation [53,54], senescence of these cells under cell culture conditions may result from lack of appropriate niche. In case of SiNSc-like and SMiNSc-like cells, the difficulties in glial differentiation may suggest their propensity toward multipotent cells, thus their infinite proliferation could not be expected.

Apparently, it should be determined whether the process of direct reprogramming generates multipotent stem cells or neural progenitors. If the first—therapeutic potential of directly reprogrammed iNSc-like cells could be increased by optimization of cell culture conditions. In case of neural progenitors generation, direct reprogramming would always be less effective than iPSCs-based method due to limited proliferation capacity of neural progenitors cells vs. neural stem cells, but still with questionable therapeutic potential for iNSCs generated *via* such method.

Undoubtedly, direct reprogramming seems to constitute a very promising approach for regenerative medicine, but still appears to be challenging in the context of human cells. On the other hand, it has been reported that mouse cells can be reprogrammed successfully with high yield [20]. It is well known that mouse cells, in particular mouse embryonic cells, undergo reprogramming more easily than human cells obtained from an adult body [20,21]. Mouse cells are in general less sensitive to replicative senescence [55]. The majority of data presented by Ring *et al*. on SOX2 direct reprogramming referred to iNSCs derived from mouse fibroblasts [7]. At the same time, the amount and quality of data on human cells appears to be insufficient. The study by Schnerch *et al*. showed that conservation of classical pluripotency factors such as OCT4/SOX2 plays an analogous role both in human and mouse iPSCs, however, downstream regulators are not conserved to the same extent [56]. Finally, mouse fibroblasts present higher proliferative potential than human fibroblasts *in vitro* [57].

## Conclusion

In the presented work we made the first attempt to compare iNc obtained with different methods. ebiNSc showed high efficiency of differentiation to neuronal and glial cells, whereas SiNSc-like and SMiNSc-like cells showed only limited features of neuronal differentiation. In contrary to iNSCs-like cells, NSC showed extremely high yield of neuronal cells generation. The potency of iNSCs-like cells direct generation was lower in comparison to iNSCs obtained by iPSCs differentiation, and only slightly improved when c-MYC was inserted. If SiNSc-like and SMiNSc-like cells proliferated, the potency of their generation would be less important. Nonetheless, SiNSc-like and SMiNSc-like cells were not able to achieve long-term survival and became senescent. Owing to this, ebiNSc seemed to constitute a better approach for therapeutic applications, since reprogramming method based on these cells is characterized by high reproducibility and high potency of iNSCs generation, ensuring high proliferative potency, self-renewal and capability of differentiation into mature, functional neuronal cells able to synthesize neurotransmitters. Therefore, iPSCs-derived neural stem cells constitute better approach, possible to be used as therapeutic tool in the near future.

## Supporting Information

S1 Table Primer sequences used for real-time PCR analysis. (PDF)

S2 Table Primary and secondary antibodies used for immunocytochemical stainings. (PDF)

S3 Table Relative expression of selected genes in analyzed cell lines. (PDF)

S4 Table Statistical data from real-time PCR analysis of selected gene expression in analyzed cell lines (Mann-Whitney U-test). Statistically significant results (p < 0.05) are shown in red.
(PDF)

## Abbreviations

NSC: neural stem cells derived from NIH-approved H9 (WA09) human embryonic stem cells
iNSCs: induced neural stem cells
iPSCs: induced pluripotent stem cells
hESCs: BG01V human embryonic stem cells
ebiNSc: induced neural stem cells obtained from iPS cells through embryoid body formation
SiNSc-like: cells that share features with induced neural stem cells obtained directly from fibroblasts by transduction with lentivirus carrying *SOX2*
SMiNSc-like: cells that share features with induced neural stem cells obtained directly from fibroblasts by transduction with lentiviruses carrying *SOX2* and *c-MYC*
iNSCs-like: cells that share features of induced neural stem cells (SiNSc-like and SMiNSc-like cells)
iNc: induced neural cells including iNSCs and iNSCs-like cells
BrdU: bromodeoxyuridine
SA-β-Gal: senescence-associated beta-galactosidase
