# Supplementary material for: SOX2 and SOX2-MYC Reprogramming Process of Fibroblasts to the Neural Stem Cells Compromised by Senescence
Source: PLoS One. 2015 Nov 4;10(11):e0141688. doi: 10.1371/journal.pone.0141688 (PMC4633175; doi:10.1371/journal.pone.0141688)
Supplement: S1 Table — (PDF) [file pone.0141688.s001.pdf]

S1 Table. Primer sequences used for real-time PCR analysis.

Primer	Sequence
HPRT1_F	5’-TGAGGATTTGGAAAGGGTGT-3’
HPRT1_R	5’-GAGCACACAGAGGGCTACAA-3’
SOX2_F	5’-GCCGAGTGGAAACTTTTGTCG-3’
SOX2_R	5’-GGCAGCGTGTACTTATCCTTCT-3’
c-MYC_F	5’-CCTGGTGCTCCATGAGGAG-3’
c-MYC-R	5’-TGGCCTCCAGCAGAAGGTGA-3’
NKX2.2_F	5’-GCACCGAGGGCCTTCAGTA-3’
NKX2.2_R	5’-GCTCGTAGGTCTGCGCCTT-3’
MSI1_F	5’-GGGACTCAGTTGGCAGACTAC-3’
MSI1_R	5’-CTGGTCCATGAAAGTGACGAA-3’
nestin_F	5’-GGAGAAACAGGGCCTACAGA-3’
nestin_R	5’-GGGAGTTCTCAGCCTCCAG-3’
COL1A1_F	5’-GAGGGCCAAGACGAAGACATC-3’
COL1A1_R	5’-CAGATCACGTCATCGCACAAC-3’
SNAI1_F	5’-TCGGAAGCCTAACTACAGCGA-3’
SNAI1_R	5’-CAGGACAGAGTCCCAGATGAGCAT-3’
TWIST2_F	5’-GCAAGAAGTCGAGCGAAGATG-3’
TWIST2_R	5’-CTCAGCTTGTCAGAGGGCAG-3’
GAD65_F	5’-GGAAACAAACTGTGCGCCCTGC-3’
GAD65_R	5’-GGTCTGTTGCATGGAGAAACGC-3’
