# Supplementary material for: SOX2 and SOX2-MYC Reprogramming Process of Fibroblasts to the Neural Stem Cells Compromised by Senescence
Source: PLoS One. 2015 Nov 4;10(11):e0141688. doi: 10.1371/journal.pone.0141688 (PMC4633175; doi:10.1371/journal.pone.0141688)
Supplement: S2 Table — (PDF) [file pone.0141688.s002.pdf]

S2 Table. Primary and secondary antibodies used for immunocytochemical stainings.

Primary antibodies used for immunocytochemical stainings			
AB I	Host	Manufacturer	Dilution
anti-SOX1	rabbit	Abcam, ab87775	1 : 100
anti-SOX2	rabbit	Millipore, AB5603	1 : 500
anti-nestin	mouse	Santa Cruz Biotechnology, Inc., sc-71665	1 : 500
anti-MAP2	rabbit	Santa Cruz Biotechnology, Inc., sc-20172	1 : 100
anti-GFAP	mouse	Millipore, MAB360	1 : 400
anti-TH	mouse	Santa Cruz Biotechnology, Inc., sc-25269	1 : 500
anti-Tau	mouse	Santa Cruz Biotechnology, Inc., sc-21796	1 : 1000
anti-Synapsin I	rabbit	Millipore, AB1543P	1 : 500
anti-VGLUT1	rabbit	Abcam, ab72311	1 : 1000
anti-αSMA	mouse	RD Systems, Mab1420	1 : 250
anti-SOX17	rabbit	Millipore, 09-038	1 : 100
**Secondary antibodies used for immunocytochemical stainings**			
AB II	Host	Manufacturer	Dilution
anti-mouse Alexa Fluor®594	donkey	Molecular Probes, Invitrogen	1 : 500
anti-rabbit Alexa Fluor®488	donkey	Molecular Probes, Invitrogen	1 : 500
