# Supplementary material for: SOX2 and SOX2-MYC Reprogramming Process of Fibroblasts to the Neural Stem Cells Compromised by Senescence
Source: PLoS One. 2015 Nov 4;10(11):e0141688. doi: 10.1371/journal.pone.0141688 (PMC4633175; doi:10.1371/journal.pone.0141688)
Supplement: S3 Table — (PDF) [file pone.0141688.s003.pdf]

S3 Table. Relative expression of selected genes in analyzed cell lines.

	SOX2	NKX2.2	MSI1	nestin	c-MYC	COL1A1	TWIST2	SNAI1
NSC/BJ	5129.573	-	15173.65	10.43304	0.229851	0.000789	0.001136	0.252094
NSC/iPSCs	13.46386	97.2572	29.76111	21.66357	0.361502	0.213031	0.291936	0.551183
NSC/ebiNSc	1.735431	66.37973	7.524457	6.37692	0.079157	0.069049	0.038039	0.30518
NSC/SiNSc-like	2.626272	5.696218	279.8485	5.487578	0.285185	0.001392	0.00074	0.205104
NSC/SMiNSc-like	3.770441	9.227946	675.6217	4.743918	0.217514	0.001835	0.000791	0.268699
ebiNSc/BJ	2955.792	-	2016.577	1.636063	2.903731	0.011427	0.029865	0.826048
ebiNSc/iPSCs	7.758222	1.465164	3.955249	3.397184	4.566901	3.085208	7.674712	1.806089
ebiNSc/NSC	0.576226	0.015065	0.1329	0.156816	12.63312	14.48245	26.28901	3.276751
ebiNSc/SiNSc-like	1.513326	0.085813	37.19185	0.860537	3.602778	0.020157	0.019466	0.672074
ebiNSc/SMiNSc-like	2.172625	0.139018	89.79008	0.74392	0.217514	0.026579	0.020797	0.880459
SiNSc-like/BJ	1953.176	-	54.22093	1.901211	0.805970	0.566924	1.534249	1.229103
SiNSc-like/iPSCs	5.126605	17.07399	0.106347	3.947748	1.267606	153.0617	394.2652	2.687337
SiNSc-like/ebiNSc	0.660796	11.6533	0.026888	1.162065	0.277564	49.61148	51.37199	1.487932
SiNSc-like/NSC	0.380768	0.175555	0.003573	0.18223	3.506494	718.4959	1350.519	4.875583
SiNSc-like/SMiNSc-like	1.435663	1.620013	2.414241	0.864483	0.762712	1.318635	1.068386	1.310062
SMiNSc-like/BJ	1360.47	-	22.45879	2.199246	1.056716	0.429933	1.436044	0.938202
SMiNSc-like/iPSCs	3.570897	10.53942	0.04405	4.5666	1.661972	116.0759	369.0287	2.051305
SMiNSc-like/ebiNSc	0.460273	7.193338	0.011137	1.344231	0.363917	37.62336	48.08372	1.135772
SMiNSc-like/SiNSc-like	0.696542	0.617279	0.414209	1.156761	1.311111	0.75836	0.935991	0.763322
SMiNSc-like/NSC	0.265221	0.108366	0.00148	0.210796	4.597403	544.8786	1264.073	3.721642
BJ/NSC	0.000195	0	6.59E-05	0.095849	4.350649	1267.358	880.2471	3.966781
BJ/iPSCs	0.002625	0	0.001961	2.076439	1.572770	269.9863	256.976	2.186421
BJ/ebiNSc	0.000338	0	0.000496	0.611223	0.344384	87.50992	33.48347	1.210583
BJ/SiNSc-like	0.000512	0	0.018443	0.525981	1.240741	1.763905	0.651784	0.813601
BJ/SMiNSc-like	0.000735	0	0.044526	0.454701	0.946328	2.325946	0.696358	1.065869
iPSCs/NSC	0.074273	0.010282	0.033601	0.04616	2.766234	4.694158	3.425406	1.81428
iPSCs/BJ	380.9883	-	509.8482	0.481594	0.635821	0.003704	0.003891	0.457368
iPSCs/ebiNSc	0.128896	0.682517	0.252829	0.294361	0.218967	0.324127	0.130298	0.553683
iPSCs/SiNSc-like	0.195061	0.058569	9.403162	0.253309	0.788889	0.006533	0.002536	0.372116

iPSCs/SMiNSc-like	0.280042	0.094882	22.7015	0.218981	0.601695	0.008615	0.00271	0.487495
