# Supplementary material for: SOX2 and SOX2-MYC Reprogramming Process of Fibroblasts to the Neural Stem Cells Compromised by Senescence
Source: PLoS One. 2015 Nov 4;10(11):e0141688. doi: 10.1371/journal.pone.0141688 (PMC4633175; doi:10.1371/journal.pone.0141688)
Supplement: S4 Table — Statistically significant results (p < 0.05) are shown in red. (PDF) [file pone.0141688.s004.pdf]

S4 Table. Statistical data from real-time PCR analysis of selected gene expression in analyzed cell lines (Mann-Whitney U-test). Statistically significant results ($p < 0.05$) are shown in red.

	p-value							
	SOX2	NKX2.2	MSI1	nestin	c-MYC	COL1A1	TWIST2	SNAI1
NSC/BJ	0.030384	0.030384	0.030384	0.030384	0.051831	0.030384	0.030384	0.030384
NSC/iPSCs	0.030384	0.030384	0.030384	0.030384	0.060603	0.665006	0.030384	0.885234
NSC/ebiNSc	0.021857	0.008475	0.008475	0.008475	0.066193	0.008475	0.008475	0.008475
NSC/SiNSc-like	0.165808	0.069955	0.014215	0.019965	0.215926	0.014215	0.030384	0.030384
NSC/SMiNSc-like	0.005821	0.028378	0.005821	0.030384	0.030384	0.005821	0.008475	0.008475
ebiNSc/BJ	0.008475	0.050799	0.008475	0.269607	0.233039	0.008475	0.008475	0.269607
ebiNSc/iPSCs	0.008475	0.798907	0.008475	0.008475	0.177911	0.074532	0.008475	0.106638
ebiNSc/SiNSc-like	0.332922	0.220032	0.002415	0.510068	0.136038	0.002415	0.008475	0.202731
ebiNSc/SMiNSc-like	0.014549	0.505161	0.000449	0.202731	0.177911	0.000449	0.000939	0.636503
SiNSc-like/BJ	0.014215	0.109820	0.240956	0.391268	0.662521	0.014215	0.193932	0.885234
SiNSc-like/iPSCs	0.749119	0.455546	0.109820	0.019965	1.000000	0.014215	0.030384	0.112352
SiNSc-like/SMiNSc-like	0.870756	0.415938	0.625484	0.391268	0.595883	0.092723	0.932324	0.552215
SMiNSc-like/BJ	0.005821	0.103877	0.005821	0.112352	0.859684	0.005821	0.148838	0.932324
SMiNSc-like/iPSCs	0.620618	0.723674	0.005821	0.030384	0.563703	0.005821	0.008475	0.106638
iPSCs/BJ	0.030384	0.030384	0.030384	0.312322	0.376760	0.030384	0.030384	0.112352
